# Rendezvous with Vaccinia Virus in the Post-smallpox Era: R&D Advances

**DOI:** 10.3390/v15081742

**Published:** 2023-08-15

**Authors:** Yuxiang Wang

**Affiliations:** Vaccine Research Center, National Institutes of Health, 40 Convent Drive, Bethesda, MD 20892, USA; yuxiang.wang@nih.gov

**Keywords:** poxvirus, vaccinia virus, smallpox, monkeypox, immunomodulation, purification, safety, innovation

## Abstract

Smallpox was eradicated in less than 200 years after Edward Jenner’s practice of cowpox variolation in 1796. The forty-three years of us living free of smallpox, beginning in 1979, never truly separated us from poxviruses. The recent outbreak of monkeypox in May 2022 might well warn us of the necessity of keeping up both the scientific research and public awareness of poxviruses. One of them in particular, the vaccinia virus (VACV), has been extensively studied as a vector given its broad host range, extraordinary thermal stability, and exceptional immunogenicity. Unceasing fundamental biological research on VACV provides us with a better understanding of its genetic elements, involvement in cellular signaling pathways, and modulation of host immune responses. This enables the rational design of safer and more efficacious next-generation vectors. To address the new technological advancement within the past decade in VACV research, this review covers the studies of viral immunomodulatory genes, modifications in commonly used vectors, novel mechanisms for rapid generation and purification of recombinant virus, and several other innovative approaches to studying its biology.

## 1. Introduction

The World Health Organization (WHO) officially announced the year 1979 as the mark of smallpox eradication [1,2]. Vaccinia virus (VACV), an orthopoxvirus belonging to the family Poxviridae and subfamily Chordopoxvirinae, was widely used as a vaccine in the eradication program and decisively contributed to the accomplishment of such extraordinary achievement. However, the manufacturing process of these smallpox vaccines at the time was not in good compliance with the standards acceptable nowadays. After all, inoculating calves with live viruses and grinding their ‘ripe’ lesions to generate a vaccine stock is neither hygienic nor quality assured [3]. Additionally, this type of vaccine contains live viruses that may lead to multiple side effects, including fever, mild rash, eczema vaccinatum, progressive vaccinia, and encephalitis, especially in immunosuppressed or immunocompromised populations [1,4,5,6]. Therefore, extensive efforts have been made to increase the safety profile of VACV, giving rise to a number of second-generation (ACAM2000 [7,8,9,10], Elstree-BN [11], and CCSV [12]), third-generation (Imvamune [13,14,15,16], NYVAC [17,18,19], and LC16m8 [20,21,22,23,24]), and next-generation smallpox vaccines (DNA vaccines [25,26,27,28], protein subunit vaccines [29,30,31], and T-cell epitope vaccines [32]) [33]. Despite the successful eradication of smallpox, poxviruses have not become a thing of the past. The potential threat of bioterrorism using the smallpox virus and the emergence or re-emergence of other poxvirus diseases should make it clear that the continuity of research is paramount. The recent outbreak of monkeypox in May 2022 is a perfect example, with more than 80,000 cases reported globally in less than a year, of which about 37.5% were identified in the US [34].

The double-stranded DNA genome of VACV has a considerable size of approximately 190 kb, containing over 200 open reading frames (ORFs). Many of these ORFs govern viral replication [35] and morphogenesis, while a significant portion also interacts extensively with the host’s antiviral immune responses. Unlike most DNA viruses, VACV forms viral factories and replicates exclusively in the cytosol to produce various types of infectious progeny. It has regained research interest largely due to unique biology: (1) inducing potent humoral and T cell-mediated immune response, (2) large genome to accommodate heterologous genes, (3) no oncogenicity for that viral genome does not integrate into host’s, (4) cost-effective manufacturing, and (5) superior thermostability for storage and logistics. The work dedicated to creating a safer smallpox vaccine not only prepared us for the outbreak of poxvirus diseases, but also begot the research using poxvirus, especially VACV, as a vector for numerous infectious diseases and oncolytic therapies. We can expect improved quality and longevity of elicited immune responses by furthering the understanding of viral genetic elements that modulate the host immune responses, regulate the expression of endogenous or exogenous genes, or determine host range. Equally important, we can anticipate achieving next-level rapid purification of recombinants through innovative techniques that not only enhance the safety profile but also facilitate convenient generation by incorporating additional mechanisms into the viral genome to control replication. Furthermore, there is never a shortage of multidisciplinary ideas for incorporating genome labeling, live imaging, and bioinformatics to study the basic biology of poxviruses. The discussion herein addresses recent technological advancements in VACV research aimed at making it a more promising vector and introduces innovations that will aid in future studies.

## 2. Enhanced Safety Mechanisms

**Figure 1:**
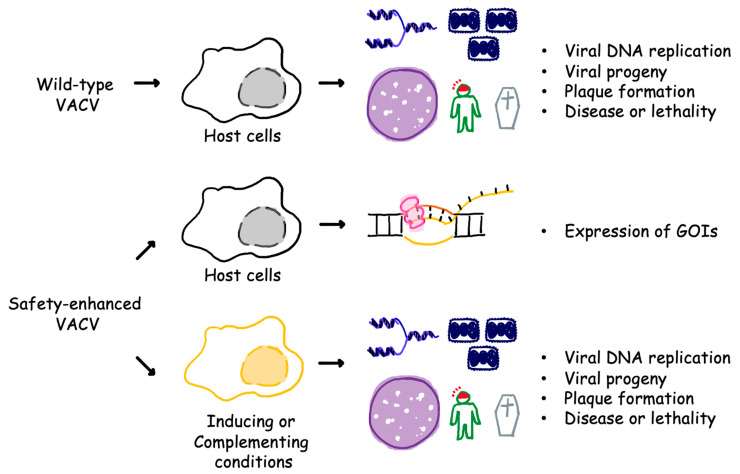
VACV with extra safety features cannot propagate in host cells. Wild-type VACVs without built-in safety mechanisms can establish an active infection. The outcomes include viral DNA replication, generation of progeny, formation of plaques, and potentially the development of diseases or death. VACVs with enhanced safety features can produce the same outcomes only if an inducing or complementing condition is provided but will not lead to an active infection in host cells. GOIs: genes of interest.

VACV effectively infects a wide range of hosts, including rodents, livestock, non-human primates, and humans. Signs of infection may range from mild fever, rash, to severe eczema vaccinatum, progressive vaccinia, and encephalitis in predisposed individuals. Highly attenuated strains, such as Modified Vaccinia Ankara (MVA), have a significantly improved safety profile due to their replication-defective nature in most mammalian cells. However, this same advantage becomes a weakness in manufacturing, as the virus does not grow to high titers, rendering the process time-consuming and cost ineffective. Generating recombinant VACVs that replicate exclusively in vitro therefore, receives extra attention. Since the replication is tightly regulated by essential genes, restricting the expression of such a gene, or conditionally supplying the expression product for a deletion mutant preferably using a stable cell line can serve as a powerful switch to control viral replication (Figure 1).

An example of conditional expression of essential genes was reported by O’Connell et al., where a tetracycline (*tet*) operon was included in a Western Reserve strain of VACV to allow the expression of viral E8R, A3L, or A6L gene only in the presence of tetracycline antibiotics [36]. The *tet* operon is derived from the transposon Tn10 operon which determines tetracycline resistance in many Gram-negative bacteria [37]. This mechanism has been extensively investigated in several model systems, including viruses, bacteria, plants, fruit flies, mammalian cells, and transgenic mice [38,39,40,41,42,43,44]. The function of VACV E8R gene has not been incontrovertibly elucidated, but studies have suggested that it is involved in the assembly process of nuclear envelope [45], is packaged into the virion [46], and might not decisively contribute to the core structure of virion [47]. The products of both A3L and A6L genes affect the morphology of virions, especially the formation of intracellular mature virions and the assembly of viral membrane [48,49]. A constitutively expressed Tet repressor protein (TetR) will bind to a *tet* operator sequence placed between the E8R, A3L, or A6L gene and its natural promoter (or a late VACV P11 promoter), immediately downstream of the transcription start site, blocking transcription in the absence of tetracycline antibiotics, such as doxycycline (DOX). An enhanced green fluorescent protein (EGFP) gene, as a transgene, was also included for the convenience of identifying plaque formation as well as abortive infections. Without DOX, two constructs viP11A3L and viP11A6L, of which the essential gene is under the control of a late VACV P11 promoter, only produced individually infected cells and failed to form plaques. This was evidenced by green fluorescence confined to each infected cell and the absence of cytopathic effects in surrounding cells. The successful expression of the transgene, independent of the generation of infectious progeny, was simultaneously confirmed. However, in the presence of DOX, the two constructs replicated to comparable titers and formed plaques that were the same size as the wild-type VACV. The safety profile was confirmed in vivo using mouse models. CB6F1/J mice when infected intranasally with viP11A6L in the absence of DOX did not develop any clinical signs or lose weight but quickly succumbed to infection when exposed to 0.125 mg/mL or higher of DOX in drinking water, just like those given wild-type VACV. No plaque-forming virions could be recovered from ovaries of mice infected intraperitoneally with viP11A6L without DOX, while a significantly higher level of viral load was detected with DOX supplied in drinking water, to a level indistinguishable from WT VACV-infected mice. Another study adapting this strategy was published by the same group to confirm the feasibility of using a replication-inducible VACV as a vaccine vector for Zika virus (vIND-ZIKV) [50]. The vaccine did not lead to any weight loss or clinical signs in intranasally infected CB6F1 mice in the absence of DOX, indicating a consistent safety profile. C57BL/6 mice vaccinated intramuscularly with a single dose of vIND-ZIKV developed high levels of ZIKV E-specific IgG and cell-mediated responses as well as being fully protected from viremia.

Coupling a replication-deficient deletion mutant with a specialized cell line that supplements the product(s) of deleted gene(s) can also greatly improve the safety profile as the vector itself is unable to replicate in vaccinees. Wyatt and colleagues reported a vector system using an A23R-deletion-mutant VACV that can only replicate in a complementing cell line [51]. The intermediate transcription factor encoded by the A23R gene is indispensable for viral replication and can be provided constitutively by complementing cells to support propagation [52,53]. To ensure high-level expression of the heterologous gene even in non-complementing cells, a T7 promoter is placed immediately upstream, and the T7 polymerase needed is expressed from a VACV early promoter in the vector. This is because viral DNA replication can still be achieved via early gene expression to generate sufficient templates containing the gene of interest. The mutant VACV could only form plaques and produce progeny in complementing RK/A8A23 cells but not in regular HeLa or RK13 cells. However, the expression of heterologous genes was not affected in BALB/c mice, evidenced by both protein expression and specific antibody responses. Eldi et al. described a similar Sementis Copenhagen Vector (SCV) system that uses the Copenhagen strain of VACV in lack of D13L gene with an engineered CHO cell line that provides D13 and CP77 proteins to allow high-titer production only in vitro [54]. D13L is responsible for the formation of infectious virions [55,56], while CP77 ensures that the replication of viral DNA is not inhibited by a cellular protein HMG20A [57]. Therefore, the genome retains the capacity to replicate in any non-permissive cell line, and high-level expression of transgenes during the late phase of the infection remains intact, but no infectious progeny can be produced. The resultant recombinant virus could not form plaques, an indicator of viral replication and cell-to-cell spread, in HEK293, MRC-5, 143b, A431, or regular CHO cell lines, but replicated to a comparable level as the replication-competent VACV in the cell substrate cell line (SCS) even after thirty passages. Electron microscopy confirmed the presence of viroplasma without immature or mature virions, suggesting arrested viral multiplication in non-permissive cell lines. An excellent safety feature was demonstrated by virulence studies in mice. Compared to severe combined immunodeficiency (SCID) mice injected with replication-competent VACV, SCV-infected ones displayed no weight loss, 100% survival rate up to 100 dpi, absence of infectious virions in multiple organs, and significantly lower clinical scores. Multiplication competence was not regained within twenty passages, as claimed by the authors.

## 3. Rapid Generation and Purification of Recombinant VACV

**Figure 2:**
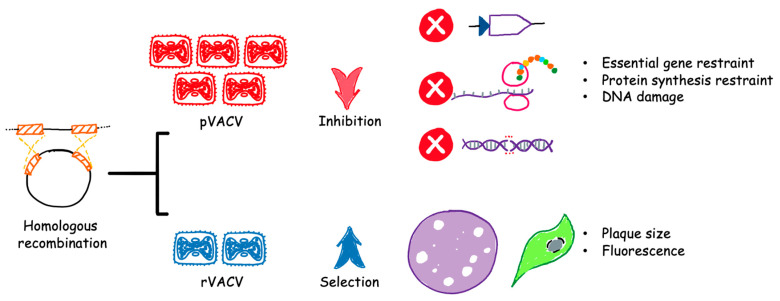
Rapid purification of recombinant VACVs is achieved by selectively suppressing the parental population. The purification process begins with homologous recombination in cells, followed by the selection of desired recombinants. Parental vaccinia viruses (pVACV) will account for a predominantly high percentage after the recombination event and need to be inhibited to accelerate the selection of recombinant vaccinia viruses (rVACV). Several major approaches to inhibition and selection are listed.

Homologous recombination has been the mainstay to introduce transgenes or deletions to VACV genome [58]. This is carried out by infecting cells with a parental virus to be genetically edited and transfecting with a shuttle plasmid that contains the gene of interest flanked by homologous sequences targeting a gene in the parental virus. However, DNA recombination is a rare event where thousands of progenies may give rise to only one desired recombinant. Therefore, a selection system is essential to distinguish between parental and recombinant viruses (Figure 2). Any system that is labor intensive or time consuming, such as transient dominant selection [59], will slow down the purification progress and reduce the flexibility of testing various genes of interest for vaccine or therapeutic purpose. On the other hand, the use of some highly efficient systems can be limited by the requirement for special equipment or reagents and cannot be widely transplanted to most laboratories. A balance between convenience and accessibility needs to be reached as innovative ideas and technologies aiming at generating recombinant VACVs advance in the future.

The difference in plaque size between parental and recombinant viruses can be a simple way for selection as reported by Siciliano et al. [60]. The parental strain vRB12 forms small plaques due to the deletion of F13L gene but will form larger plaques upon its restoration by a plasmid that also encodes the gene of interest [60]. However, distinguishing between large and small plaques is not a clear binary system, and parental viruses can be easily carried over, leading to the need for repetitive and additional purification. Cao and co-workers constructed a double-deletion mutant that can only propagate in permissive cell lines to simplify the selection of recombinants [61]. The E3L and K3L genes in the parental virus are, respectively, interrupted by an EGFP and a mCherry gene. In the shuttle vector, homology arms targeting the K3L gene in the parental virus flank a taterapox virus K3L ortholog and a heterologous gene of interest. Homologous recombination occurs in permissive HeLa/PKR knockout or A549/PKR+Rnase L knockout cells and subsequent purification happens in non-permissive BHK21 cells. Nearly all green plaques are successful recombinants. Despite accelerated purification, this system still relies on a fluorescent protein marker. An EPPIC (Efficient Purification by Parental Inducer Constraint) system independent of a selection marker was developed by Jasperse et al. to achieve both generation and purification of recombinant VACVs within one week [62], using minimal equipment available in any lab setting. Taking advantage of their previously reported replication-inducible system [36], a lactose-inducible (replicates only in the presence of isopropyl β-D-1-thiogalactopyranoside, or commonly known as IPTG) parental virus undergoes homologous recombination with a shuttle vector containing gene(s) of interest to give rise to a tet-inducible (replicates only in the presence of DOX) recombinant virus. Since the inducing conditions for parental and recombinant viruses are mutually exclusive, removing IPTG and adding DOX to the culture media will make it solely conducive to the growth of recombinant viruses. Cells infected by parental viruses will not show any cytopathic effect, enabling easy isolation and sequential purification. This is particularly important for making marker-free vectors as fluorescent protein genes, despite convenience in selecting for recombinant virus, cannot be present in a vaccine candidate. Similar to this idea, White and co-workers added a negative selection marker to inhibit viral replication in parental virus to greatly increase the percent of recombinants [63]. The inhibition is achieved by blocking protein synthesis in infected cells via coumermycin-activated phosphorylation of translation initiation factor eIF2α. The coumermycin-binding domain is derived from Escherichia coli gyrase B (GyrB) and is fused to a mammalian double-stranded RNA-dependent protein kinase PKR [64]. In the presence of the antibiotic coumermycin which binds to two molecules of GyrB, dimerization of GyrB will occur and activate PKR catalytic domain to phosphorylate eIF2α. This system significantly decreased the ratio of parental to recombinant viruses from 4000:1 (without coumermycin) to 1:80 (with coumermycin). Although distinguishing between parental and recombinant viruses is more straightforward, the necessity for multiple rounds of plaque purification remains.

Several other approaches to generating marker-free recombinant VACV have also been explored. Introducing a fluorescent marker during recombination then cleaving it using Cre/loxP system [65,66] once successful recombinants are purified is a strategy used in Rintoul and co-workers’ work [67]. They placed loxP sites on both sides of the fluorescence gene which is flanked by homology arms specific to the VACV genome. Isolation of recombinant plaques could be achieved by identifying fluorescence and facilitated using fluorescence-activated cell sorting (FACS). Purified recombinant viruses were then grown in a cell line that expresses Cre recombinase to excise the marker gene. The same strategy was also applied in the work of Guo et al. to generate multiple versions of VACV vectors expressing a human C-C motif chemokine ligand 5 (CCL5) or cytokines [68]. A next-level technique to accelerate the generation and purification of recombinant VACV is CRISPR/Cas9 system [69,70,71,72]. It is an undeniably popular gene editing tool in multiple living organisms [73,74,75,76,77,78,79,80]. Yuan et al., in their two studies [69,71], increased the efficiency of homologous recombination by expressing the Cas9 endonuclease gene from Streptococcus pyogenes and single-guide RNA in VACV infected cells co-transfected with a repair plasmid that contains a cassette of red fluorescent protein (RFP) marker gene and a tumor antigen TRP2 flanked by homology arms targeting a VACV gene. Modification of two VACV genes simultaneously was also confirmed using this method. A follow-up study of theirs sought to remove the RFP marker gene by adding loxP sites on both sides, so that infected cells expressing Cre recombinase would excise the RFP gene [71]. However, this strategy did not address the challenge of isolating red plaques from a background overwhelmingly populated by colorless plaques of parental viruses. An elaborated version of this approach by Gowripalan and co-workers [70] provided an explanation and evidence for the limited improvement in homologous recombination rate by the CRISPR/Cas9 system as, in spite of the efficient DNA cleavage by Cas9, neither of the two repairing pathways (non-homologous end joining or homology-directed repair) was highly functional. Instead, they took advantage of the inefficient DNA repair in parental VACV to render them replication deficient, which in turn significantly increased the chance of isolating recombinant virus. After introducing Cas9 and single-guide RNA that targets a fluorescence gene (only present in parental viruses) as ribonucleoprotein complexes to a mixture that contained just 5% of successfully recombined viruses, this percentage surged to 80%. With a second round of editing, nearly all residual parental viruses were eliminated. A parallel experiment in this study tested the feasibility of abandoning the fluorescent marker and yielded a similar increase in the purity of recombinant virus from 4% to 71% with just two rounds of Cas9 selection. A further extension of combining CRISPR/Cas9 with trimolecular recombination was reported by Laudermilch and Chandran, termed MAVERICC (marker-free vaccinia virus engineering of recombinants through in vitro CRISPR/Cas9 cleavage) [72]. This strategy utilized cell-free viral DNA cleavage by Cas9/sgRNA to first generate cleaved VACV DNA before joining it with helper virus (fowlpox virus) infected cells co-transfected with a rescue amplicon that contains the transgene flanked by homologous regions specific to cleaved parental VACV DNA. In a single step, without the need for sequential purification, both wild type and recombinant viruses could be rescued after trimolecular recombination [81]. Among the randomly selected plaques, recombinant viruses accounted for a stunning 91% (10 out of 11 plaques for a truncation mutant), 100% (12 out of 12 plaques for a K151E mutant), or 92% (11 out of 12 plaques for an insertion mutant). Simultaneous modifications in two genes were also tested in this study and showed a similarly high percentage (70%, 7 out of 10) of desired recombinants in the rescued viruses.

## 4. Modification in Viral Immunomodulatory Genes

Vaccinia virus has a large genome that includes a plethora of immunomodulatory genes interfering with host antiviral immune responses [82,83]. For example, the complement system is targeted by a viral protein VCP that functions to cleave cellular C3b and C4b [84,85]. VACV is also well known to inhibit the production and binding of several interferons (IFN), chemokines, and cytokines. As a vector bearing genes of interest, one would hope that the characteristics of the vector would enhance, rather than diminish, host immunity specific to the target antigen. Therefore, immunomodulatory genes in VACV may exert negative effects on desired immune responses, and deleting or modifying these genes has become a promising strategy to improve the immunogenicity of VACV vectors.

To quantitatively evaluate the effects of such a gene on antigen-specific immune responses after immunization, it is common to first generate a deletion mutant identical to the parental virus in every way but the presence of the immunomodulatory gene of interest. Analysis of growth kinetics in several permissive cell lines, such as chick embryo fibroblasts in vitro, will follow to confirm if the deleted gene is essential for viral replication, which can be reflected by plaque formation and titer. An infection experiment will also be conducted in human macrophages and dendritic cells to compare the mRNA and protein levels of gene expression involved in innate immunity, including interleukins (IL), IFN-β, interferon-induced proteins with tetratricopeptide repeats (IFIT), melanoma differentiation-associated protein 5 (MDA-5), tumor necrosis factor alpha (TNF-α), and macrophage inflammatory protein-1 alpha (MIP-1α). Subsequent in vivo studies will focus on evaluating antigen-specific adaptive immune responses during both the primary and memory phases. Taking the HIV-1 vaccine with DNA priming and VACV boosting as an example, CD4+ and CD8+ T-cell as well as antibody responses have been extensively investigated, to address the potential improvements resulting from the deletion of immune modulators in the vector. The secretion of IFN-γ, TNF-α, IL-2, and surface expression of CD107a by T cells upon peptide stimulation, targeting HIV Gag, Env, and Gag-Pol-Nef fusion proteins, can well represent the magnitude, breadth, and polyfunctionality of antigen-specific immune responses. Total IgG responses against HIV-1 gp120 protein and the VACV vector serve as another arm for quantification, revealing the quality of immune responses in the absence of the vaccinia immunomodulatory gene.

Multiple VACV genes have gained particular interest. The A40R gene is involved in the early phase of infection and is reported to play a role in virulence [86] or is an essential gene [87]. It encodes a type II membrane glycoprotein found on the surface of infected cells [88], but it has also been shown to form a complex with a small SUMO-1 peptide, which is believed to aid in viral replication [87]. The A46R gene encodes a viral protein containing a Toll-interleukin receptor (TIR) domain which binds to adaptor molecules involved in Toll-like receptor (TLR) signaling pathways [88], functioning essentially as a TLR signaling inhibitor. A41L is known to counteract the function of CC-chemokines by reducing their concentration instead of antagonizing the receptor binding process [89]. A52R is involved in disrupting several TLR signaling pathways by blocking the activation of nuclear factor kappa B (NF-κB) [90]. The expression product of B2R has nuclease activity and destabilizes the cellular cyclic GMP-AMP (2′3′ cGAMP) messenger that is involved in stimulator of interferon genes (STING)-dependent antiviral responses [91]. B7R encodes a soluble decoy for TNF and binds specifically to several chemokines [92]. B8 is a soluble glycoprotein binding and inhibiting a wide spectrum of IFN-γ [93]. B9R produces a small intracellular protein responsible for viral replication in vivo [94]. B13R is an early gene, and its product is known to prevent apoptosis in infected cells [95]. B15R (equivalent to B14 in the Western Reserve strain) encodes an intracellular virulence factor that disrupts TLR signaling by inhibiting I kappa B (IκB) kinase [96]. A soluble IL-1β receptor is expressed by B16R and interferes with the acute phase of innate immunity [97]. B19 is a soluble glycoprotein that broadly binds to type I IFNs from a wide host range [98]. C6L is an early gene that serves as an IFN-β inhibitor to block interferon regulatory factor (IRF) signaling [99]. C7L is a viral host range gene, and its expression product is anti-apoptotic, inhibiting type I interferons [100,101,102]. C12L codes for an IL-18-binding protein that blocks IFN-γ expression [103]. E5R encodes a virulence factor that also directly inhibits cellular cyclic GMP-AMP synthase (cGAS) to evade the host cGAS/STING pathway [104]. K7R is a member of the VACV Bcl-2 family and upregulates histone methylation as well as blocks TLR signaling by inhibiting NF-κB and IRF3 activation [105,106,107]. N2L also belongs to the Bcl-2 family, functioning as an inhibitor of TLR signaling pathways [107,108].

A list of reported attempts to delete immunomodulatory genes in VACV vectors over the past ten years can be seen in Table 1 (not exhaustive). However, enhanced immune responses do not always result from the generation of deletion mutants, largely due to inadequate knowledge of gene functions and the complexity of systematic interactions, especially when multiple genes are deleted. An example of improved immunogenicity was reported in a study by Pérez and co-workers on an HIV-1 vaccine candidate based on an A40R-deleted MVA [109]. They showed that A40 protein is not required for viral growth in vitro, as the deletion mutant grew to similar titers as the parental virus in DF-1 cells. In the absence of A40R gene, the mRNA levels of IFN-β, IFIT1&2, MDA-5, and MIP-1α significantly increased in human THP-1 cells, except for IFN-β, which showed an increase at 3 h post-infection (hpi), while the others showed an increase at 6 hpi. The magnitude of HIV-1-specific primary CD4+ and CD8+ T-cell responses were, respectively, 2.3- and 1.4-fold higher in mice boosted with the deletion mutant. Additionally, the polyfunctionality of antigen-specific T cells was improved, as 90% of stimulated CD4+ T cells expressed at least two types of cytokines or surface markers with CD107a-IFN-γ-TNF-α-IL-2- and IFN-γ-TNF-α-IL-2-populations being the dominant ones. CD8+ T cells were also polyfunctional upon stimulation with 80% of them displaying at least two hallmarks. The most abundant populations were CD107a-IFN-γ-TNF-α and CD107a-IFN-γ. Elevated memory T-cell responses were detected almost 8 weeks post boost vaccination, with twice as high percentages of antigen-specific CD4+ and CD8+ T cells in mice that received the deletion mutant. These memory T cells exhibited polyfunctionality similar to those in primary responses. Particularly, during both the primary and memory phases, the deletion of the A40R gene elicited a remarkably higher percentage of T effector memory and T effector cells. Quantitation of total anti-gp120 IgG (IgG1, IgG2a, and IgG3) revealed a significant increase in humoral responses in the absence of A40R gene both 10 and 53 days post-boost. Contrary to the phenotypes reported above, Marín et al. developed an MVA based hepatitis C virus vaccine candidate with C6L gene deletion but did not obtain comparably improved immune responses [110]. Despite similar growth kinetics to the parental virus without C6L deletion in DF-1 cells, the mutant still indistinguishably downregulated IFN-β, IFIT1&2, and TNF-α in infected human macrophages and monocyte-derived dendritic cells. The levels of HCV-specific CD8+ T-cell responses were essentially unchanged upon deletion during both primary and memory phases except that the deletion mutant was more NS3 specific while the parental virus was more p7 and NS2 specific. Anti-E2 and anti-vector total IgG responses were also similar.

## 5. Other Aspects of Innovation

Information can become overwhelming if disorganized, and it is particularly true for research on VACV vectors. Even within the scope of this article, the extensive discussion on safety mechanisms, rapid purification of recombinant VACVs, and modifications in viral immunomodulatory genes will need to be updated in the next few years. Identifying and locating new reports on construction and application of VACV vectors can be time consuming. The search results may be inexhaustive and new experiments based on the incomplete list may lead to repeatedly produced data. A user-friendly database that can be conveniently updated should unquestionably receive more attention. An example of a web-based database, named Vaxvec, for recombinant vaccine vectors was reported by Deng and co-workers [126]. It falls under the umbrella of VIOLIN (Vaccine Investigation and Online Information Network), the first platform that includes thousands of vaccines and hundreds of diseases [127,128] but provides additional information specifically for vaccine vectors and antigens of interest in these recombinant vectors. For instance, a page displaying recombinant vectors will include links to all vaccines that use the vector as well as the protective antigens. The authors mentioned that the addition of immune responses to this database will tailor this platform to the booming research on vectored vaccine development.

We still have a long way to go to understand the VACV genome. The very fact that viral replication occurs intracellularly and is hard to track necessitates an advancement in labeling methods. Luminescence imaging can serve as a powerful tool to visualize infection in cells or live animals, e.g., the expression product of a luciferase reporter gene inserted into the VACV genome can react with substrates available in cell culture or circulation in an animal and emit light that can be captured [129]. Similarly, Kieser and co-workers combined live time-lapse fluorescence microscopy with DNA labeling to track the replication and recombination events of VACV [130]. The bacteriophage lambda DNA-binding protein, cro, when fused with a fluorescent protein such as GFP, can nonspecifically bind to DNA and therefore locate any cytoplasmic DNA during infection. Another DNA labeling technology, ANCHOR, was adopted by Gallardo et al. to evaluate VACV infection kinetics by inserting to its genome an ANCH sequence and an OR3-Santaka sequence [131]. The ANCH sequence in viral DNA copies provides a docking site for OR-Santaka fluorescent protein which oligomerizes upon binding to this sequence. Live-cell imaging can then detect the behaviors of labeled viral DNA particles in the cytoplasm and reveal dynamic viral replication. This fluorescent tagging signal remained strong even after cell fixation and other processing procedures used in common staining methods. However, early replication steps with minimal protein involvement can pose a challenge to this approach. To address this limit, Mok et al. demonstrated a tracking system using a nucleoside analog, 5-ethynyl-2′-deoxyuridine (EdU), that can locate single viral genomes in replication factories or even when they are still packed in the viral core [132]. An azide (N3^−^)-containing fluorescent dye can then covalently form a heteroatom ring with the nucleoside analog for easy visualization, and this process is not sensitive to crowded cytoplasmic compartments due to the involvement of only small molecules.

The research and development of novel VACV vectors admittedly draw the most attention, but the manufacturing and delivery of vaccines also deserve recognition. To replace chick embryo fibroblasts (CEF), which are primary cells with a limited lifespan, new permanent cell lines have been generated for MVA production in bioreactors, including cell lines derived from ducks, quails, and monkeys [133,134,135,136]. Jordan and colleagues reported a biphasic suspension culture system, scalable to 200-L bioreactors, aiming for high cell density during phase 1, while promoting aggregation when a chemical inducer is added in phase 2 to facilitate viral spread and growth [137]. A titer higher than 10^8^ PFU/mL of viruses was achieved using just a few million cells [138]. Chen et al. reported two new freeze-drying formulations to significantly increase the storage time of a VACV vectored vaccine without compromising antigen stability [139]. The vaccine was mixed with a formula consisting of polyethylene glycol, dextran, bovine serum albumin, and L-glutamic acid at different ratios, lyophilized, and kept at either 4 °C or 25 °C for up to four weeks. Notwithstanding that all formulae similarly preserved titer at 4 °C for four weeks, with the maximal loss being less than 30-fold even for PBS-formulated vaccine, only two of the five formulae managed to limit the loss of titer at 25 °C to less than 100-fold, while the other groups had a nearly million-fold decrease. The antigen-specific T-cell and antibody responses induced by the formulated vaccines were indistinguishable from those of the non-freeze-drying control.

The majority of research involving VACV vectors as vaccines is currently in the preclinical phase. Some address bacterial infections, such as leishmaniasis [124], malaria [140], and tuberculosis [141,142]. Most focus on viral diseases of clinical relevance. A wide array of vaccine research centers around HIV-1 [109,116,121,143,144,145,146,147,148,149,150,151,152,153,154,155,156,157,158,159,160]. Plentiful studies target arboviruses that are on the rise: dengue virus [161], Zika virus [50,162,163,164,165], West Nile virus [166], yellow fever virus [167], tick-borne encephalitis virus [168,169], chikungunya virus [163,170], and Crimean-Congo hemorrhagic fever virus [171]. Multiple diseases of high prevalence or fatality rate, especially those with epidemic or pandemic potential garner particular attention, including the following examples: ebolavirus [172,173,174,175], Nipah virus [176], influenza virus [177,178,179,180], respiratory syncytial virus [181,182], Middle East respiratory syndrome coronavirus [183,184], and needless to say, severe acute respiratory syndrome coronavirus 2 (SARS-CoV-2) [158,185,186,187,188,189,190,191,192,193,194,195]. Several VACV-vectored vaccines have entered clinical trials: HIV-1 [196], tuberculosis [197,198], respiratory syncytial virus [199], influenza A virus [200], and SARS-CoV-2 [201]. Worldwide oral rabies vaccination programs, which aim to control the spread of rabies in its natural reservoir, also utilize recombinant VACV expressing the rabies virus glycoprotein gene (e.g., RABORAL V-RG) [202]. Licensed vaccines based on VACV are now available not only to prevent orthopoxvirus infection but also for various other infectious diseases. Recommended by the Advisory Committee on Immunization Practices (ACIP), JYNNEOS has become an alternative to ACAM2000 as Monkeypox vaccine, effective since November 2021. MVA-BN-Filo (Mvabea) is a multivalent vaccine against Ebola Virus Disease (EVD), administered in a two-dose regimen along with an adenovirus-vectored vaccine [203]. It encodes the glycoprotein gene from several different viruses: Ebola virus Mayinga, Sudan virus (SUDV) Gulu, and Marburg (MARV) virus Musoke. The nucleoprotein gene of Tai Forest virus (TAFV) is also included.

## 6. Future Perspectives

Vaccinia virus has been long known to us since its use as a vaccine to eradicate smallpox. The advancement of genetic and immunologic research not only improved the safety profile of VACV as a smallpox vaccine but also as a recombinant vector [204]. A multitude of approaches to finely controlling viral replication by introducing inducible systems or complementing cell lines yielded exciting results in animal studies. Innovative methods to accelerate the generation of recombinant VACVs considerably reduced the turnover time to obtain and test a desired phenotype. Deepening the understanding of viral gene functions offered the opportunity to bring vector modification to the next level for a more immunogenic and protective vaccine or therapeutic vector. The participation of multidisciplinary efforts enabled the rational design of new VACV vectors with easily accessible data support, the exposure and monitoring of previously hidden replication events using novel labeling and imaging techniques, and the cost-efficient manufacture and distribution of vaccines beyond the R&D stage.

We are at the dawn of explosive technological advancement and should welcome changes to traditional research practices as well. Artificial intelligence (AI) integrated platforms may well mark the beginning of a new era of literature review and data management. For example, ChatGPT is a chatbot developed by OpenAI. Since its release in November 2022, billions of users have been amazed by its outstanding performance. It can handle computer coding work, consolidate information, write poems and songs, and take tests or exams. Limitations do exist, such as providing ostensible answers that are factually nonsensical. However, we cannot neglect the potential of incorporating AI into scientific research, including in the development of VACV-vectored vaccines. Imagine an AI-assisted or even AI-operated platform that collects all publications on VACV by exhaustion and categorizes them by topic or field. Critical findings and conclusions are grouped and cross-compared to return a pattern of research interests shared among researchers. Input from new studies, such as identification of gene functions, mapping of epitopes, elucidation of immune responses, inter alia, can be conveniently added in a user-friendly manner. Redundancy in similar study designs will be minimized, if not eliminated altogether, and the consolidation of information in one comprehensive view should help us shift from reductionist biology to systems biology in the next step. If the process of literature review could transition from a brick-and-mortar library to the Internet around two decades ago, the same transition should be embraced now with the advent of AI.

High-throughput automation is no longer a dream, even in a small laboratory, as the exorbitant prices of robots for PCR, ELISA, imaging, etc., are no longer an obstacle. One can anticipate the streamlined full spectrum of VACV research in most lab settings. The generation and purification of recombinant viruses will be handled automatically in 96-well or 384-well microplates in addition to the built-in genetic modifications that ensure predominant populations of desired recombinants. All possible combinations of gene deletion or modification can be tested in vitro before modeling in animals, after which the automatic characterization of T-cell and antibody responses would also be achieved. The viral infection and replication will be visualized and imaged live with innovative DNA labeling to allow for high-resolution monitoring at every step involved in these events. “A picture is worth a thousand words, then a video has to be worth at least 1.8 million words”, says McQuivey in “How Video Will Take Over the World”. While live images of biological events provide unprecedented insights into the interactions between biomacromolecules, prolonged dynamic imaging, enabled by automation, can transform a picture into a video, capturing the trajectory of molecules and adding a temporal axis.

Incessant investigations into the biology and immunology of VACV, coupled with technological innovations in automation, centralized information management, high-throughput purification, cost-efficient manufacturing, and optimized formulation, are now rejuvenating this ‘ancient’ virus that once made it possible to claim victory against smallpox. Its path is now converging with ours yet again and will surely bear more fruit in the future.

## Figures and Tables

**Table 1:** VACV immunomodulatory genes investigated in deletion mutants for vaccine research.

VACV Genes	Functions	Mutants Reported in Vaccine Research
A40R	Virulence [86] or essential gene [87,111]	[109]
A41L	Chemokine inhibitor [89]	[112,113]
A46R	NF-κB inhibitor [88]	[113,114,115,116]
A52R	NF-κB inhibitor [90]	[116,117,118]
B2R	IFN and NF-κB inhibitor [91]	[119] *
B6R	Unknown	[116]
B7R	TNF decoy [92]	[116]
B8R	IFN-γ inhibitor [93]	[116,120]
B9R	Replication in vivo [94]	[116]
B10R	Unknown	[116]
B13R	Apoptosis inhibitor [95]	[121]
B15R	IκB kinase inhibitor [96]	[113,116,117,118]
B16R	IL-1β decoy [97]	[112]
B19R	Type I IFN inhibitor [98]	[116,120]
C6L	IFN-β inhibitor [99]	[110,122,123]
C7L	Type I IFN inhibitor [100,101,102]	[124]
C12L	IL-18 inhibitor [103]	[113,115]
E5R	Virulence factor and IFN inhibitor [104]	[104]
K7R	NF-κB inhibitor [105,106,107]	[116,117,118,123]
N2L	IRF3 inhibitor [107,108]	[125]

* An antitumor study utilizing the oncolytic feature of VACV.

## Data Availability

Not applicable.

## References

[1] Fenner F., Henderson D.A., Arita I., Ježek Z., Ladnyi I.D. (1988). Smallpox and Its Eradication.

[2] Wehrle P.F. (1980). A reality in our time—Certification of the global eradication of smallpox. J. Infect. Dis..

[3] Esparza J., Lederman S., Nitsche A., Damaso C.R. (2020). Early smallpox vaccine manufacturing in the United States: Introduction of the “animal vaccine” in 1870, establishment of “vaccine farms”, and the beginnings of the vaccine industry. Vaccine.

[4] Lane J.M., Ruben F.L., Neff J.M., Millar J.D. (1970). Complications of smallpox vaccination, 1968: Results of ten statewide surveys. J. Infect. Dis..

[5] Lane J.M., Goldstein J. (2003). Evaluation of 21st-century risks of smallpox vaccination and policy options. Ann. Intern. Med..

[6] Kemper A.R., Davis M.M., Freed G.L. (2002). Expected adverse events in a mass smallpox vaccination campaign. Eff. Clin. Pract..

[7] Weltzin R., Liu J., Pugachev K.V., Myers G.A., Coughlin B., Blum P.S., Nichols R., Johnson C., Cruz J., Kennedy J.S. (2003). Clonal vaccinia virus grown in cell culture as a new smallpox vaccine. Nat. Med..

[8] Monath T.P., Caldwell J.R., Mundt W., Fusco J., Johnson C.S., Buller M., Liu J., Gardner B., Downing G., Blum P.S. (2004). ACAM2000 clonal Vero cell culture vaccinia virus (New York City Board of Health strain)—A second-generation smallpox vaccine for biological defense. Int. J. Infect. Dis..

[9] Frey S.E., Newman F.K., Kennedy J.S., Ennis F., Abate G., Hoft D.F., Monath T.P. (2009). Comparison of the safety and immunogenicity of ACAM1000, ACAM2000 and Dryvax in healthy vaccinia-naive adults. Vaccine.

[10] Greenberg R.N., Kennedy J.S. (2008). ACAM2000: A newly licensed cell culture-based live vaccinia smallpox vaccine. Expert. Opin. Investig. Drugs.

[11] Stittelaar K.J., Amerongen G., van Kondova I., Kuiken T., Lavieren R.F., van Pistoor F.H.M., Niesters H.G.M., van Doornum G., van der Zeijst B.A.M., Mateo L. (2005). Modified Vaccinia Virus Ankara Protects Macaques against Respiratory Challenge with Monkeypox Virus. J. Virol..

[12] Greenberg R.N., Kennedy J.S., Clanton D.J., Plummer E.A., Hague L., Cruz J., Ennis F.A., Blackwelder W.C., Hopkins R.J. (2005). Safety and immunogenicity of new cell-cultured smallpox vaccine compared with calf-lymph derived vaccine: A blind, single-centre, randomised controlled trial. Lancet.

[13] von Krempelhuber A., Vollmar J., Pokorny R., Rapp P., Wulff N., Petzold B., Handley A., Mateo L., Siersbol H., Kollaritsch H. (2010). A randomized, double-blind, dose-finding Phase II study to evaluate immunogenicity and safety of the third generation smallpox vaccine candidate IMVAMUNE. Vaccine.

[14] Vollmar J., Arndtz N., Eckl K.M., Thomsen T., Petzold B., Mateo L., Schlereth B., Handley A., King L., Hülsemann V. (2006). Safety and immunogenicity of IMVAMUNE, a promising candidate as a third generation smallpox vaccine. Vaccine.

[15] Drexler I., Heller K., Wahren B., Erfle V., Sutter G. (1998). Highly attenuated modified vaccinia virus Ankara replicates in baby hamster kidney cells, a potential host for virus propagation, but not in various human transformed and primary cells. J. Gen. Virol..

[16] Mayr A., Stickl H., Müller H.K., Danner K., Singer H. (1978). The smallpox vaccination strain MVA: Marker, genetic structure, experience gained with the parenteral vaccination and behavior in organisms with a debilitated defence mechanism (author’s transl). Zentralbl. Bakteriol. B.

[17] Midgley C.M., Putz M.M., Weber J.N., Smith G.L. (2008). Vaccinia virus strain NYVAC induces substantially lower and qualitatively different human antibody responses compared with strains Lister and Dryvax. J. Gen. Virol..

[18] Paoletti E., Tartaglia J., Taylor J. (1994). Safe and effective poxvirus vectors—NYVAC and ALVAC. Dev. Biol. Stand..

[19] Tartaglia J., Perkus M.E., Taylor J., Norton E.K., Audonnet J.C., Cox W.I., Davis S.W., Van Der Hoeven J., Meignier B., Riviere M. (1992). NYVAC: A highly attenuated strain of vaccinia virus. Virology.

[20] Johnson B.F., Kanatani Y., Fujii T., Saito T., Yokote H., Smith G.L. (2011). Serological responses in humans to the smallpox vaccine LC16m8. J. Gen. Virol..

[21] Kenner J., Cameron F., Empig C., Jobes D.V., Gurwith M. (2006). LC16m8: An attenuated smallpox vaccine. Vaccine.

[22] Takahashi-Nishimaki F., Funahashi S.I., Miki K., Hashizume S., Sugimoto M. (1991). Regulation of plaque size and host range by a vaccinia virus gene related to complement system proteins. Virology.

[23] Kennedy J.S., Gurwith M., Dekker C.L., Frey S.E., Edwards K.M., Kenner J., Lock M., Empig C., Morikawa S., Saijo M. (2011). Safety and Immunogenicity of LC16m8, an Attenuated Smallpox Vaccine in Vaccinia-Naive Adults. J. Infect. Dis..

[24] Saito T., Fujii T., Kanatani Y., Saijo M., Morikawa S., Yokote H., Takeuchi T., Kuwabara N. (2009). Clinical and Immunological Response to Attenuated Tissue-Cultured Smallpox Vaccine LC16m8. JAMA.

[25] Hirao L.A., Draghia-Akli R., Prigge J.T., Yang M., Satishchandran A., Wu L., Hammarlund E., Khan A.S., Babas T., Rhodes L. (2011). Multivalent Smallpox DNA Vaccine Delivered by Intradermal Electroporation Drives Protective Immunity in Nonhuman Primates Against Lethal Monkeypox Challenge. J. Infect. Dis..

[26] Pulford D.J., Gates A., Bridge S.H., Robinson J.H., Ulaeto D. (2004). Differential efficacy of vaccinia virus envelope proteins administered by DNA immunisation in protection of BALB/c mice from a lethal intranasal poxvirus challenge. Vaccine.

[27] Sakhatskyy P., Wang S., Zhang C., Chou T.H., Kishko M., Lu S. (2008). Immunogenicity and Protection Efficacy of Subunit-based Smallpox Vaccines Using Variola Major Antigens. Virology.

[28] Hooper J.W., Custer D.M., Thompson E. (2003). Four-gene-combination DNA vaccine protects mice against a lethal vaccinia virus challenge and elicits appropriate antibody responses in nonhuman primates. Virology.

[29] Buchman G.W., Cohen M.E., Xiao Y., Richardson-Harman N., Silvera P., DeTolla L.J., Davis H.L., Eisenberg R.J., Cohen G.H., Isaacs S.N. (2010). A protein-based smallpox vaccine protects non-human primates from a lethal monkeypox virus challenge. Vaccine.

[30] Davies D.H., McCausland M.M., Valdez C., Huynh D., Hernandez J.E., Mu Y., Hirst S., Villarreal L., Felgner P.L., Crotty S. (2005). Vaccinia virus H3L envelope protein is a major target of neutralizing antibodies in humans and elicits protection against lethal challenge in mice. J. Virol..

[31] Fogg C., Lustig S., Whitbeck J.C., Eisenberg R.J., Cohen G.H., Moss B. (2004). Protective immunity to vaccinia virus induced by vaccination with multiple recombinant outer membrane proteins of intracellular and extracellular virions. J. Virol..

[32] Moise L., Buller R.M., Schriewer J., Lee J., Frey S.E., Weiner D.B., Martin W., De Groot A.S. (2011). VennVax, a DNA-prime, peptide-boost multi-T-cell epitope poxvirus vaccine, induces protective immunity against vaccinia infection by T cell response alone. Vaccine.

[33] Verardi P.H., Titong A., Hagen C.J. (2012). A vaccinia virus renaissance: New vaccine and immunotherapeutic uses after smallpox eradication. Hum. Vaccin. Immunother..

[34] Centers for Disease Control and Prevention 2022 Outbreak Cases and Data|Mpox|Poxvirus. https://www.cdc.gov/poxvirus/mpox/response/2022/index.html.

[35] Liu B., Panda D., Mendez-Rios J.D., Ganesan S., Wyatt L.S., Moss B. (2018). Identification of Poxvirus Genome Uncoating and DNA Replication Factors with Mutually Redundant Roles. J. Virol..

[36] O’Connell C.M., Jasperse B., Hagen C.J., Titong A., Verardi P.H. (2020). Replication-inducible vaccinia virus vectors with enhanced safety in vivo. PLoS ONE.

[37] Hillen W., Berens C. (1994). Mechanisms underlying expression of Tn10 encoded tetracycline resistance. Annu. Rev. Microbiol..

[38] Zhu Z., Zheng T., Lee C.G., Homer R.J., Elias J.A. (2002). Tetracycline-controlled transcriptional regulation systems: Advances and application in transgenic animal modeling. Semin. Cell Dev. Biol..

[39] Yao F., Svensjö T., Winkler T., Lu M., Eriksson C., Eriksson E. (1998). Tetracycline repressor, tetR, rather than the tetR-mammalian cell transcription factor fusion derivatives, regulates inducible gene expression in mammalian cells. Hum. Gene Ther..

[40] Stieger K., Belbellaa B., le Guiner C., Moullier P., Rolling F. (2009). In vivo gene regulation using tetracycline-regulatable systems. Adv. Drug Deliv. Rev..

[41] Stebbins M.J., Urlinger S., Byrne G., Bello B., Hillen W., Yin J.C.P. (2001). Tetracycline-inducible systems for Drosophila. Proc. Natl. Acad. Sci. USA.

[42] Gatz C., Quail P.H. (1988). Tn10-encoded tet repressor can regulate an operator-containing plant promoter. Proc. Natl. Acad. Sci. USA.

[43] Faryar K., Gatz C. (1992). Construction of a tetracycline-inducible promoter in Schizosaccharomyces pombe. Curr. Genet..

[44] Bertram R., Hillen W. (2008). The application of Tet repressor in prokaryotic gene regulation and expression. Microb. Biotechnol..

[45] Tolonen N., Doglio L., Schleich S., Krijnse Locker J. (2001). Vaccinia virus DNA replication occurs in endoplasmic reticulum-enclosed cytoplasmic mini-nuclei. Mol. Biol. Cell.

[46] Doglio L., de Marco A., Schleich S., Roos N., Krijnse Locker J. (2002). The Vaccinia virus E8R gene product: A viral membrane protein that is made early in infection and packaged into the virions’ core. J. Virol..

[47] Kato S.E.M., Condit R.C., Moussatché N. (2007). The vaccinia virus E8R gene product is required for formation of transcriptionally active virions. Virology.

[48] Kato S.E.M., Strahl A.L., Moussatche N., Condit R.C. (2004). Temperature-sensitive mutants in the vaccinia virus 4b virion structural protein assemble malformed, transcriptionally inactive intracellular mature virions. Virology.

[49] Moss B. (2015). Poxvirus membrane biogenesis. Virology.

[50] Jasperse B., O’Connell C.M., Wang Y., Verardi P.H. (2021). Single dose of a replication-defective vaccinia virus expressing Zika virus-like particles is protective in mice. Sci. Rep..

[51] Wyatt L.S., Xiao W., Americo J.L., Earl P.L., Moss B. (2017). Novel Nonreplicating Vaccinia Virus Vector Enhances Expression of Heterologous Genes and Suppresses Synthesis of Endogenous Viral Proteins. MBio.

[52] Sanz P., Moss B. (1999). Identification of a transcription factor, encoded by two vaccinia virus early genes, that regulates the intermediate stage of viral gene expression. Proc. Natl. Acad. Sci. USA.

[53] Warren R.D., Cotter C.A., Moss B. (2012). Reverse Genetics Analysis of Poxvirus Intermediate Transcription Factors. J. Virol..

[54] Eldi P., Cooper T.H., Liu L., Prow N.A., Diener K.R., Howley P.M., Suhrbier A., Hayball J.D. (2017). Production of a Chikungunya Vaccine Using a CHO Cell and Attenuated Viral-Based Platform Technology. Mol. Ther..

[55] Liu L., Cooper T., Howley P.M., Hayball J.D. (2014). From crescent to mature virion: Vaccinia virus assembly and maturation. Viruses.

[56] Zhang Y., Moss B. (1992). Immature viral envelope formation is interrupted at the same stage by lac operator-mediated repression of the vaccinia virus D13L gene and by the drug rifampicin. Virology.

[57] Hsiao J.C., Chao C.C., Young M.J., Chang Y.T., Cho E.C., Chang W. (2006). A poxvirus host range protein, CP77, binds to a cellular protein, HMG20A, and regulates its dissociation from the vaccinia virus genome in CHO-K1 cells. J. Virol..

[58] Wyatt L.S., Earl P.L., Moss B. (2017). Generation of Recombinant Vaccinia Viruses. Curr. Protoc. Protein Sci..

[59] Falkner F.G., Moss B. (1990). Transient dominant selection of recombinant vaccinia viruses. J. Virol..

[60] Siciliano N.A., Huang L., Eisenlohr L.C. (2013). Recombinant poxviruses: Versatile tools for immunological assays. Methods Mol. Biol..

[61] Cao J., Layne C., Varga J., Deschambault Y. (2020). Application of poxvirus K3 ortholog as a positive selection marker for constructing recombinant vaccinia viruses with modified host range. MethodsX.

[62] Jasperse B., O’Connell C.M., Wang Y., Verardi P.H. (2020). EPPIC (Efficient Purification by Parental Inducer Constraint) Platform for Rapid Generation of Recombinant Vaccinia Viruses. Mol. Ther. Methods Clin. Dev..

[63] White S.D., Conwell K., Langland J.O., Jacobs B.L. (2011). Use of a negative selectable marker for rapid selection of recombinant vaccinia virus. Biotechniques.

[64] Ung T.L., Cao C., Lu J., Ozato K., Dever T.E. (2001). Heterologous dimerization domains functionally substitute for the double-stranded RNA binding domains of the kinase PKR. EMBO J..

[65] Sternberg N., Hamilton D. (1981). Bacteriophage P1 site-specific recombination. I. Recombination between loxP sites. J. Mol. Biol..

[66] Sauer B., Henderson N. (1988). Site-specific DNA recombination in mammalian cells by the Cre recombinase of bacteriophage P1. Proc. Natl. Acad. Sci. USA.

[67] Rintoul J.L., Wang J., Gammon D.B., van Buuren N.J., Garson K., Jardine K., Barry M., Evans D.H., Bell J.C. (2011). A selectable and excisable marker system for the rapid creation of recombinant poxviruses. PLoS ONE.

[68] Guo Z.S., Liu Z., Sathaiah M., Wang J., Ravindranathan R., Kim E., Huang S., Kenniston T.W., Bell J.C., Zeh H.J. (2017). Rapid Generation of Multiple Loci-Engineered Marker-free Poxvirus and Characterization of a Clinical-Grade Oncolytic Vaccinia Virus. Mol. Ther. Methods Clin. Dev..

[69] Yuan M., Zhang W., Wang J., Al Yaghchi C., Ahmed J., Chard L., Lemoine N.R., Wang Y. (2015). Efficiently Editing the Vaccinia Virus Genome by Using the CRISPR-Cas9 System. J. Virol..

[70] Gowripalan A., Smith S., Stefanovic T., Tscharke D.C. (2020). Rapid poxvirus engineering using CRISPR/Cas9 as a selection tool. Commun. Biol..

[71] Yuan M., Gao X., Chard L.S., Ali Z., Ahmed J., Li Y., Liu P., Lemoine N.R., Wang Y. (2015). A marker-free system for highly efficient construction of vaccinia virus vectors using CRISPR Cas9. Mol. Ther. Methods Clin. Dev..

[72] Laudermilch E., Chandran K. (2021). MAVERICC: Marker-free vaccinia virus engineering of recombinants through in vitro CRISPR/Cas9 cleavage. J. Mol. Biol..

[73] Borca M.V., Holinka L.G., Berggren K.A., Gladue D.P. (2018). CRISPR-Cas9, a tool to efficiently increase the development of recombinant African swine fever viruses. Sci. Rep..

[74] Bi Y., Sun L., Gao D., Ding C., Li Z., Li Y., Cun W., Li Q. (2014). High-efficiency targeted editing of large viral genomes by RNA-guided nucleases. PLoS Pathog..

[75] Russell T.A., Stefanovic T., Tscharke D.C. (2015). Engineering herpes simplex viruses by infection-transfection methods including recombination site targeting by CRISPR/Cas9 nucleases. J. Virol. Methods.

[76] Yang H., Wang H., Shivalila C.S., Cheng A.W., Shi L., Jaenisch R. (2013). One-step generation of mice carrying reporter and conditional alleles by CRISPR/Cas-mediated genome engineering. Cell.

[77] Jiang W., Bikard D., Cox D., Zhang F., Marraffini L.A. (2013). RNA-guided editing of bacterial genomes using CRISPR-Cas systems. Nat. Biotechnol..

[78] Shi T.Q., Liu G.N., Ji R.Y., Shi K., Song P., Ren L.J., Huang H., Ji X.-J. (2017). CRISPR/Cas9-based genome editing of the filamentous fungi: The state of the art. Appl. Microbiol. Biotechnol..

[79] Lander N., Chiurillo M.A., Docampo R. (2016). Genome Editing by CRISPR/Cas9: A Game Change in the Genetic Manipulation of Protists. J. Eukaryot. Microbiol..

[80] Ebrahimi S., Teimoori A., Khanbabaei H., Tabasi M. (2019). Harnessing CRISPR/Cas 9 System for manipulation of DNA virus genome. Rev. Med. Virol..

[81] Smith E.S., Shi S., Zauderer M. (2004). Construction of cDNA libraries in vaccinia virus. Methods Mol. Biol..

[82] Smith G.L., Benfield C.T.O., Maluquer de Motes C., Mazzon M., Ember S.W.J., Ferguson B.J., Sumner R.P. (2013). Vaccinia virus immune evasion: Mechanisms, virulence and immunogenicity. J. Gen. Virol..

[83] Albarnaz J.D., Torres A.A., Smith G.L. (2018). Modulating Vaccinia Virus Immunomodulators to Improve Immunological Memory. Viruses.

[84] Kotwal G.J., Moss B. (1988). Vaccinia virus encodes a secretory polypeptide structurally related to complement control proteins. Nature.

[85] McKenzie R., Kotwal G.J., Moss B., Hammer C.H., Frank M.M. (1992). Regulation of complement activity by vaccinia virus complement-control protein. J. Infect. Dis..

[86] Tscharke D.C., Reading P.C., Smith G.L. (2002). Dermal infection with vaccinia virus reveals roles for virus proteins not seen using other inoculation routes. J. Gen. Virol..

[87] Palacios S., Perez L.H., Welsch S., Schleich S., Chmielarska K., Melchior F., Krijnse Locker J. (2005). Quantitative SUMO-1 modification of a vaccinia virus protein is required for its specific localization and prevents its self-association. Mol. Biol. Cell.

[88] Wilcock D., Duncan S.A., Traktman P., Zhang W.H., Smith G.L. (1999). The vaccinia virus A4OR gene product is a nonstructural, type II membrane glycoprotein that is expressed at the cell surface. J. Gen. Virol..

[89] Stack J., Haga I.R., Schröder M., Bartlett N.W., Maloney G., Reading P.C., Fitzgerald K.A., Smith G.L., Bowie A.G. (2005). Vaccinia virus protein A46R targets multiple Toll-like–interleukin-1 receptor adaptors and contributes to virulence. J. Exp. Med..

[90] Bahar M.W., Kenyon J.C., Putz M.M., Abrescia N.G.A., Pease J.E., Wise E.L., Stuart D.I., Smith G.L., Grimes J.M. (2008). Structure and function of A41, a vaccinia virus chemokine binding protein. PLoS Pathog..

[91] Harte M.T., Haga I.R., Maloney G., Gray P., Reading P.C., Bartlett N.W., Smith G.L., Bowie A., O’Neill L.A.J. (2003). The Poxvirus Protein A52R Targets Toll-like Receptor Signaling Complexes to Suppress Host Defense. J. Exp. Med..

[92] Eaglesham J.B., Pan Y., Kupper T.S., Kranzusch P.J. (2019). Viral and metazoan poxins are cGAMP-specific nucleases that restrict cGAS-STING signaling. Nature.

[93] Alejo A., Ruiz-Argüello M.B., Ho Y., Smith V.P., Saraiva M., Alcami A. (2006). A chemokine-binding domain in the tumor necrosis factor receptor from variola (smallpox) virus. Proc. Natl. Acad. Sci. USA.

[94] Symons J.A., Tscharke D.C., Price N., Smith G.L. (2002). A study of the vaccinia virus interferon-gamma receptor and its contribution to virus virulence. J. Gen. Virol..

[95] Price N., Tscharke D.C., Smith G.L. (2002). The vaccinia virus B9R protein is a 6 kDa intracellular protein that is non-essential for virus replication and virulence. J. Gen. Virol..

[96] Dobbelstein M., Shenk T. (1996). Protection against apoptosis by the vaccinia virus SPI-2 (B13R) gene product. J. Virol..

[97] Chen R.A.J., Ryzhakov G., Cooray S., Randow F., Smith G.L. (2008). Inhibition of IκB Kinase by Vaccinia Virus Virulence Factor B14. PLoS Pathog..

[98] Staib C., Kisling S., Erfle V., Sutter G. (2005). Inactivation of the viral interleukin 1beta receptor improves CD8+ T-cell memory responses elicited upon immunization with modified vaccinia virus Ankara. J. Gen. Virol..

[99] Symons J.A., Alcamí A., Smith G.L. (1995). Vaccinia virus encodes a soluble type I interferon receptor of novel structure and broad species specificity. Cell.

[100] Unterholzner L., Sumner R.P., Baran M., Ren H., Mansur D.S., Bourke N.M., Randow F., Smith G.L., Bowie A.G. (2011). Vaccinia Virus Protein C6 Is a Virulence Factor that Binds TBK-1 Adaptor Proteins and Inhibits Activation of IRF3 and IRF7. PLoS Pathog..

[101] Perkus M.E., Goebel S.J., Davis S.W., Johnson G.P., Limbach K., Norton E.K., Paoletti E. (1990). Vaccinia virus host range genes. Virology.

[102] Nájera J.L., Gómez C.E., Domingo-Gil E., Gherardi M.M., Esteban M. (2006). Cellular and biochemical differences between two attenuated poxvirus vaccine candidates (MVA and NYVAC) and role of the C7L gene. J. Virol..

[103] Meng X., Jiang C., Arsenio J., Dick K., Cao J., Xiang Y. (2009). Vaccinia virus K1L and C7L inhibit antiviral activities induced by type I interferons. J. Virol..

[104] Smith V.P., Bryant N.A., Alcamí A. (2000). Ectromelia, vaccinia and cowpox viruses encode secreted interleu-kin-18-binding proteins. J. Gen. Virol..

[105] Yang N., Wang Y., Dai P., Li T., Zierhut C., Tan A., Zhang T., Xiang J.Z., Ordureau A., Funabiki H. (2023). Vaccinia E5 is a major inhibitor of the DNA sensor cGAS. Nat. Commun..

[106] Schröder M., Baran M., Bowie A.G. (2008). Viral targeting of DEAD box protein 3 reveals its role in TBK1/IKKɛ-mediated IRF activation. EMBO J..

[107] Teferi W.M., Desaulniers M.A., Noyce R.S., Shenouda M., Umer B., Evans D.H. (2017). The vaccinia virus K7 protein promotes histone methylation associated with heterochromatin formation. PLoS ONE.

[108] Gonzlez J.M., Esteban M. (2010). A poxvirus Bcl-2-like gene family involved in regulation of host immune response: Sequence similarity and evolutionary history. Virol. J..

[109] Ferguson B.J., Benfield C.T.O., Ren H., Lee V.H., Frazer G.L., Strnadova P., Sumner R.P., Smith G.L. (2013). Vaccinia virus protein N2 is a nuclear IRF3 inhibitor that promotes virulence. J. Gen. Virol..

[110] Pérez P., Marín M.Q., Lázaro-Frías A., Sorzano C.Ó.S., Gómez C.E., Esteban M., García-Arriaza J. (2020). Deletion of Vaccinia Virus A40R Gene Improves the Immunogenicity of the HIV-1 Vaccine Candidate MVA-B. Vaccines.

[111] Marín M.Q., Pérez P., Gómez C.E., Sorzano C.Ó.S., Esteban M., García-Arriaza J. (2018). Removal of the C6 Vaccinia Virus Interferon-β Inhibitor in the Hepatitis C Vaccine Candidate MVA-HCV Elicited in Mice High Immunogenicity in Spite of Reduced Host Gene Expression. Viruses.

[112] García-Arriaza J., Nájera J.L., Gómez C.E., Sorzano C.O.S., Esteban M. (2010). Immunogenic Profiling in Mice of a HIV/AIDS Vaccine Candidate (MVA-B) Expressing Four HIV-1 Antigens and Potentiation by Specific Gene Deletions. PLoS ONE.

[113] Garber D.A., O’Mara L.A., Gangadhara S., McQuoid M., Zhang X., Zheng R., Gill K., Verma M., Yu T., Johnson B. (2012). Deletion of Specific Immune-Modulatory Genes from Modified Vaccinia Virus Ankara-Based HIV Vaccines Engenders Improved Immunogenicity in Rhesus Macaques. J. Virol..

[114] Perdiguero B., Gómez C.E., Di Pilato M., Sorzano C.O.S., Delaloye J., Roger T., Calandra T., Pantaleo G., Esteban M. (2013). Deletion of the Vaccinia Virus Gene A46R, Encoding for an Inhibitor of TLR Signalling, Is an Effective Approach to Enhance the Immunogenicity in Mice of the HIV/AIDS Vaccine Candidate NYVAC-C. PLoS ONE.

[115] Holgado M.P., Falivene J., Maeto C., Amigo M., Pascutti M.F., Vecchione M.B., Bruttomesso A., Calamante G., Del Médico-Zajac M.P., Gherardi M.M. (2016). Deletion of A44L, A46R and C12L Vaccinia Virus Genes from the MVA Genome Improved the Vector Immunogenicity by Modifying the Innate Immune Response Generating Enhanced and Optimized Specific T-Cell Responses. Viruses.

[116] Gómez C.E., Perdiguero B., Sánchez-Corzo C., Sorzano C.O.S., Esteban M. (2018). Immune Modulation of NYVAC-Based HIV Vaccines by Combined Deletion of Viral Genes that Act on Several Signalling Pathways. Viruses.

[117] Di Pilato M., Mejías-Pérez E., Zonca M., Perdiguero B., Gómez C.E., Trakala M., Nieto J., Nájera J.L., SSorzano C.O., Combadière C. (2015). NFκB activation by modified vaccinia virus as a novel strategy to enhance neutrophil migration and HIV-specific T-cell responses. Proc. Natl. Acad. Sci. USA.

[118] Di Pilato M., Mejías-Pérez E., Sorzano C.O., Esteban M. (2017). Distinct Roles of Vaccinia Virus NF-κB Inhibitor Proteins A52, B15, and K7 in the Immune Response. J. Virol..

[119] Riederer S., del Canizo A., Navas J., Peter M.G., Link E.K., Sutter G., Rojas J.J. (2023). Improving poxvirus-mediated antitumor immune responses by deleting viral cGAMP-specific nuclease. Cancer Gene Ther..

[120] Gomez C.E., Perdiguero B., Najera J.L., Sorzano C.O.S., Jimenez V., Gonzalez-Sanz R., Esteban M. (2012). Removal of Vaccinia Virus Genes That Block Interferon Type I and II Pathways Improves Adaptive and Memory Responses of the HIV/AIDS Vaccine Candidate NYVAC-C in Mice. J. Virol..

[121] Chea L.S., Wyatt L.S., Gangadhara S., Moss B., Amara R.R. (2019). Novel Modified Vaccinia Virus Ankara Vector Expressing Anti-apoptotic Gene B13R Delays Apoptosis and Enhances Humoral Responses. J. Virol..

[122] García-Arriaza J., Nájera J.L., Gómez C.E., Tewabe N., Sorzano C.O.S., Calandra T., Roger T., Esteban M. (2011). A candidate HIV/AIDS vaccine (MVA-B) lacking vaccinia virus gene C6L enhances memory HIV-1-specific T-cell responses. PLoS ONE.

[123] García-Arriaza J., Arnáez P., Gómez C.E., Sorzano C.Ó.S., Esteban M. (2013). Improving Adaptive and Memory Immune Responses of an HIV/AIDS Vaccine Candidate MVA-B by Deletion of Vaccinia Virus Genes (C6L and K7R) Blocking Interferon Signaling Pathways. PLoS ONE.

[124] Sánchez-Sampedro L., Mejías-Pérez E., SSorzano C.Ó., Nájera J.L., Esteban M. (2016). NYVAC vector modified by C7L viral gene insertion improves T cell immune responses and effectiveness against leishmaniasis. Virus Res..

[125] Garcia-Arriaza J., Gomez C.E., Sorzano C.O.S., Esteban M. (2014). Deletion of the Vaccinia Virus N2L Gene Encoding an Inhibitor of IRF3 Improves the Immunogenicity of Modified Vaccinia Virus Ankara Expressing HIV-1 Antigens. J. Virol..

[126] Deng S., Martin C., Patil R., Zhu F., Zhao B., Xiang Z., He Y. (2015). Vaxvec: The first web-based recombinant vaccine vector database and its data analysis. Vaccine.

[127] He Y., Racz R., Sayers S., Lin Y., Todd T., Hur J., Li X., Patel M., Zhao B., Chung M. (2014). Updates on the web-based VIOLIN vaccine database and analysis system. Nucleic Acids Res..

[128] Xiang Z., Todd T., Ku K.P., Kovacic B.L., Larson C.B., Chen F., Hodges A.P., Tian Y., Olenzek E.A., Zhao B. (2008). VIOLIN: Vaccine investigation and online information network. Nucleic Acids Res..

[129] Perdiguero B., Gómez C.E., Esteban M. (2019). Bioluminescence Imaging as a Tool for Poxvirus Biology. Methods in Molecular Biology.

[130] Kieser Q., Paszkowski P., Lin J., Evans D., Noyce R. (2019). Visualizing Poxvirus Replication and Recombination Using Live-Cell Imaging. Methods Mol. Biol..

[131] Gallardo F., Schmitt D., Brandely R., Brua C., Silvestre N., Findeli A., Foloppe J., Top S., Kappler-Gratias S., Quentin-Froignant C. (2020). Fluorescent Tagged Vaccinia Virus Genome Allows Rapid and Efficient Measurement of Oncolytic Potential and Discovery of Oncolytic Modulators. Biomedicines.

[132] Mok H., Yakimovich A. (2019). Click Chemistry-Based Labeling of Poxvirus Genomes. Methods Mol. Biol..

[133] Jordan I., Vos A., Beilfuß S., Neubert A., Breul S., Sandig V. (2009). An avian cell line designed for production of highly attenuated viruses. Vaccine.

[134] Léon A., David A.L., Madeline B., Guianvarc’h L., Dureau E., Champion-Arnaud P., Hebben M., Huss T., Chatrenet B., Schwamborn K. (2016). The EB66^®^ cell line as a valuable cell substrate for MVA-based vaccines production. Vaccine.

[135] Kraus B., Fircks S., von Feigl S., Koch S.M., Fleischanderl D., Terler K., Dersch-Pourmojib M., Konetschny C., Grillberger L., Reiter M. (2011). Avian cell line—Technology for large scale vaccine production. BMC Proc..

[136] Mayr A. (2004). Genetically Engineered Virus for Use in the Propagation of Preferential Cells. U.S. Patent.

[137] Jordan I., Lohr V., Genzel Y., Reichl U., Sandig V. (2013). Elements in the Development of a Production Process for Modified Vaccinia Virus Ankara. Microorganisms.

[138] Jordan I., Northoff S., Thiele M., Hartmann S., Horn D., Höwing K., Bernhardt H., Oehmke S., von Horsten H., Rebeski D. (2011). A chemically defined production process for highly attenuated poxviruses. Biologicals.

[139] Chen Y., Liao Q., Chen T., Zhang Y., Yuan W., Xu J., Zhang X. (2021). Freeze-Drying Formulations Increased the Adenovirus and Poxvirus Vaccine Storage Times and Antigen Stabilities. Virol. Sin..

[140] Hou M.M., Barrett J.R., Themistocleous Y., Rawlinson T.A., Diouf A., Martinez F.J., Nielsen C.M., Lias A.M., King L.D.W., Edwards N.J. (2023). Vaccination with Plasmodium vivax Duffy-binding protein inhibits parasite growth during controlled human malaria infection. Sci. Transl. Med..

[141] Leung-Theung-Long S., Gouanvic M., Coupet C.A., Ray A., Tupin E., Silvestre N., Marchand J.B., Schmitt D., Hoffmann C., Klein M. (2015). A Novel MVA-Based Multiphasic Vaccine for Prevention or Treatment of Tuberculosis Induces Broad and Multifunctional Cell-Mediated Immunity in Mice and Primates. PLoS ONE.

[142] Nangpal P., Bahal R.K., Tyagi A.K. (2017). Boosting with recombinant MVA expressing M. tuberculosis α-crystallin antigen augments the protection imparted by BCG against tuberculosis in guinea pigs. Sci. Rep..

[143] García-Arriaza J., Perdiguero B., Heeney J.L., Seaman M.S., Montefiori D.C., Yates N.L., Tomaras G.D., Ferrari G., Foulds K.E., Roederer M. (2017). HIV/AIDS Vaccine Candidates Based on Replication-Competent Recombinant Poxvirus NYVAC-C-KC Expressing Trimeric gp140 and Gag-Derived Virus-Like Particles or Lacking the Viral Molecule B19 That Inhibits Type I Interferon Activate Relevant HIV-1-Specific B and T Cell Immune Functions in Nonhuman Primates. J. Virol..

[144] Chege G.K., Burgers W.A., Müller T.L., Gray C.M., Shephard E.G., Barnett S.W., Ferrari G., Montefiori D., Williamson C., Williamson A.L. (2017). DNA-MVA-protein vaccination of rhesus macaques induces HIV-specific immunity in mucosal-associated lymph nodes and functional antibodies. Vaccine.

[145] Bradley T., Pollara J., Santra S., Vandergrift N., Pittala S., Bailey-Kellogg C., Shen X., Parks R., Goodman D., Eaton A. (2017). Pentavalent HIV-1 vaccine protects against simian-human immunodeficiency virus challenge. Nat. Commun..

[146] Saunders K.O., Santra S., Parks R., Yates N.L., Sutherland L.L., Scearce R.M., Balachandran H., Bradley T., Goodman D., Eaton A. (2018). Immunogenicity of NYVAC Prime-Protein Boost Human Immunodeficiency Virus Type 1 Envelope Vaccination and Simian-Human Immunodeficiency Virus Challenge of Nonhuman Primates. J. Virol..

[147] Asbach B., Kibler K.V., Köstler J., Perdiguero B., Yates N.L., Stanfield-Oakley S., Tomaras G.D., Kao S.F., Foulds K.E., Roederer M. (2019). Priming with a Potent HIV-1 DNA Vaccine Frames the Quality of Immune Responses prior to a Poxvirus and Protein Boost. J. Virol..

[148] Perdiguero B., Gómez C.E., García-Arriaza J., Sánchez-Corzo C., Sorzano C.Ó.S., Wilmschen S., von Laer D., Asbach B., Schmalzl C., Peterhoff D. (2019). Heterologous Combination of VSV-GP and NYVAC Vectors Expressing HIV-1 Trimeric gp145 Env as Vaccination Strategy to Induce Balanced B and T Cell Immune Responses. Front. Immunol..

[149] Raman S.C., Mejías-Pérez E., Gomez C.E., García-Arriaza J., Perdiguero B., Vijayan A., Pérez-Ruiz M., Cuervo A., Santiago C., Sorzano C.O.S. (2019). The Envelope-Based Fusion Antigen GP120C14K Forming Hexamer-Like Structures Triggers T Cell and Neutralizing Antibody Responses Against HIV-1. Front. Immunol..

[150] Lévy Y., Lacabaratz C., Ellefsen-Lavoie K., Stöhr W., Lelièvre J.D., Bart P.A., Launay O., Weber J., Salzberger B., Wiedemann A. (2020). Optimal priming of poxvirus vector (NYVAC)based HIV vaccine regimens for T cell responses requires three DNA injections. Results of the randomized multicentre EV03/ ANRS VAC20 Phase I/II Trial. PLoS Pathog..

[151] Gómez C.E., Perdiguero B., Usero L., Marcos-Villar L., Miralles L., Leal L., Sorzano C.Ó.S., Sánchez-Corzo C., Plana M., García F. (2021). Enhancement of the HIV-1-Specific Immune Response Induced by an mRNA Vaccine through Boosting with a Poxvirus MVA Vector Expressing the Same Antigen. Vaccines.

[152] Wee E.G., Moyo N., Hannoun Z., Giorgi E.E., Korber B., Hanke T. (2021). Effect of epitope variant co-delivery on the depth of CD8 T cell responses induced by HIV-1 conserved mosaic vaccines. Mol. Ther. Methods Clin. Dev..

[153] Yu J., Li Y., Zhong M., Yang J., Zhou D., Zhao B., Cao Y., Yan H., Zhang E., Yang Y. (2018). Improved immune response against HIV-1 Env antigen by enhancing EEV production via a K151E mutation in the A34R gene of replication-competent vaccinia virus Tiantan. Antiviral Res..

[154] Kibler K.V., Asbach B., Perdiguero B., García-Arriaza J., Yates N.L., Parks R., Stanfield-Oakley S., Ferrari G., Montefiori D.C., Tomaras G.D. (2019). Replication-Competent NYVAC-KC Yields Improved Immunogenicity to HIV-1 Antigens in Rhesus Macaques Compared to Nonreplicating NYVAC. J. Virol..

[155] Pérez P., Marín M.Q., Lázaro-Frías A., Sorzano C.Ó.S., Di Pilato M., Gómez C.E., Esteban M., García-Arriaza J. (2019). An MVA Vector Expressing HIV-1 Envelope under the Control of a Potent Vaccinia Virus Promoter as a Promising Strategy in HIV/AIDS Vaccine Design. Vaccines.

[156] Bollimpelli V.S., Reddy P.B.J., Gangadhara S., Charles T.P., Burton S.L., Tharp G.K., Styles T.M., Labranche C.C., Smith J.C., Upadhyay A.A. (2023). Intradermal but not intramuscular modified vaccinia Ankara immunizations protect against intravaginal tier2 simian-human immunodeficiency virus challenges in female macaques. Nat. Commun..

[157] Falqui M., Perdiguero B., Coloma R., Albert M., Marcos-Villar L., McGrail J.P., Sorzano C.Ó.S., Esteban M., Gómez C.E., Guerra S. (2023). An MVA-based vector expressing cell-free ISG15 increases IFN-I production and improves HIV-1-specific CD8 T cell immune responses. Front. Cell Infect. Microbiol..

[158] Lorenzo M.M., Marín-López A., Chiem K., Jimenez-Cabello L., Ullah I., Utrilla-Trigo S., Calvo-Pinilla E., Lorenzo G., Moreno S., Ye C. (2023). Vaccinia Virus Strain MVA Expressing a Prefusion-Stabilized SARS-CoV-2 Spike Glycoprotein Induces Robust Protection and Prevents Brain Infection in Mouse and Hamster Models. Vaccines.

[159] Styles T.M., Gangadhara S., Reddy P.B.J., Sahoo A., Shiferaw A., Welbourn S., Kozlowski P.A., Derdeyn C.A., Velu V., Amara R.R. (2022). V2 hotspot optimized MVA vaccine expressing stabilized HIV-1 Clade C envelope Gp140 delays acquisition of heterologous Clade C Tier 2 challenges in Mamu-A*01 negative Rhesus Macaques. Front. Immunol..

[160] Sahoo A., Jones A.T., Cheedarla N., Gangadhara S., Roy V., Styles T.M., Shiferaw A., Walter K.L., Williams L.T.D., Shen X. (2022). A clade C HIV-1 vaccine protects against heterologous SHIV infection by modulating IgG glycosylation and T helper response in macaques. Sci. Immunol..

[161] Wilken L., Stelz S., Agac A., Sutter G., Prajeeth C.K., Rimmelzwaan G.F. (2023). Recombinant Modified Vaccinia Virus Ankara Expressing a Glycosylation Mutant of Dengue Virus NS1 Induces Specific Antibody and T-Cell Responses in Mice. Vaccines.

[162] Pérez P., Marín M.Q., Lázaro-Frías A., Jiménez de Oya N., Blázquez A.B., Escribano-Romero E., Carlos C.Ó., Ortego J., Saiz J.C., Esteban M. (2018). A Vaccine Based on a Modified Vaccinia Virus Ankara Vector Expressing Zika Virus Structural Proteins Controls Zika Virus Replication in Mice. Sci. Rep..

[163] Prow N.A., Liu L., McCarthy M.K., Walters K., Kalkeri R., Geiger J., Koide F., Cooper T.H., Eldi P., Nakayama E. (2020). The vaccinia virus based Sementis Copenhagen Vector vaccine against Zika and chikungunya is immunogenic in non-human primates. npj Vaccines.

[164] Zhan Y., Deng Y., Huang B., Song Q., Wang W., Yang Y., Dai L., Wang W., Yan J., Gao G.F. (2019). Humoral and cellular immunity against both ZIKV and poxvirus is elicited by a two-dose regimen using DNA and non-replicating vaccinia virus-based vaccine candidates. Vaccine.

[165] Jiménez de Oya N., Pérez P., Blázquez A.B., Escribano-Romero E., Esteban M., Saiz J.C., García-Arriaza J., Mar-tín-Acebes M.A. (2022). Low Immune Cross-Reactivity between West Nile Virus and a Zika Virus Vaccine Based on Modified Vaccinia Virus Ankara. Pharmaceuticals.

[166] Volz A., Lim S., Kaserer M., Lülf A., Marr L., Jany S., Deeg C.A., Pijlman G.P., Koraka P., Osterhaus A.D.M.E. (2016). Immunogenicity and protective efficacy of recombinant Modified Vaccinia virus Ankara candidate vaccines delivering West Nile virus envelope antigens. Vaccine.

[167] Julander J.G., Testori M., Cheminay C., Volkmann A. (2018). Immunogenicity and protection after vaccination with a modified vaccinia virus Ankara-vectored yellow fever vaccine in the hamster model. Front. Immunol..

[168] Beicht J., Kubinski M., Zdora I., Puff C., Biermann J., Gerlach T., Baumgärtner W., Sutter G., Osterhaus A.D.M.E., Prajeeth C.K. (2023). Induction of humoral and cell-mediated immunity to the NS1 protein of TBEV with recombinant Influenza virus and MVA affords partial protection against lethal TBEV infection in mice. Front. Immunol..

[169] Kubinski M., Beicht J., Zdora I., Biermann J., Puff C., Gerlach T., Tscherne A., Baumgärtner W., Osterhaus A.D.M.E., Sutter G. (2023). A recombinant Modified Vaccinia virus Ankara expressing prME of tick-borne encephalitis virus affords mice full protection against TBEV infection. Front. Immunol..

[170] García-Arriaza J., Esteban M., López D. (2021). Modified Vaccinia Virus Ankara as a Viral Vector for Vaccine Candidates against Chikungunya Virus. Biomedicines.

[171] Buttigieg K.R., Dowall S.D., Findlay-Wilson S., Miloszewska A., Rayner E., Hewson R., Carroll M.W. (2014). A novel vaccine against Crimean-Congo haemorrhagic fever protects 100% of animals against lethal challenge in a mouse model. PLoS ONE.

[172] Malherbe D.C., Domi A., Hauser M.J., Atyeo C., Fischinger S., Hyde M.A., Williams J.M., Alter G., Guirakhoo F., Bukreyev A. (2022). A single immunization with a modified vaccinia Ankara vectored vaccine producing Sudan virus-like particles protects from lethal infection. NPJ Vaccines.

[173] Lázaro-Frías A., Gómez-Medina S., Sánchez-Sampedro L., Ljungberg K., Ustav M., Liljeström P., Muñoz-Fontela C., Esteban M., García-Arriaza J. (2018). Distinct Immunogenicity and Efficacy of Poxvirus-Based Vaccine Candidates against Ebola Virus Expressing GP and VP40 Proteins. J. Virol..

[174] Rahim M.N., Wee E.G., He S., Audet J., Tierney K., Moyo N., Hannoun Z., Crook A., Baines A., Korber B. (2019). Complete protection of the BALB/c and C57BL/6J mice against Ebola and Marburg virus lethal challenges by pan-filovirus T-cell epigraph vaccine. PLoS Pathog..

[175] Xie L., Zai J., Yi K., Li Y. (2019). Intranasal immunization with recombinant Vaccinia virus Tiantan harboring Zaire Ebola virus gp elicited systemic and mucosal neutralizing antibody in mice. Vaccine.

[176] Medina-Magües E.S., Lopera-Madrid J., Lo M.K., Spiropoulou C.F., Montgomery J.M., Medina-Magües L.G., Salas-Quinchucua C., Jiménez-Mora A.P., Osorio J.E. (2023). Immunogenicity of poxvirus-based vaccines against Nipah virus. Sci. Rep..

[177] Coughlan L., Sridhar S., Payne R., Edmans M., Milicic A., Venkatraman N., Lugonja B., Clifton L., Qi C., Folegatti P.M. (2018). Heterologous Two-Dose Vaccination with Simian Adenovirus and Poxvirus Vectors Elicits Long-Lasting Cellular Immunity to Influenza Virus A in Healthy Adults. EBioMedicine.

[178] Langenmayer M.C., Luelf-Averhoff A.T., Marr L., Jany S., Freudenstein A., Adam-Neumair S., Tscherne A., Fux R., Rojas J.J., Blutke A. (2023). Newly Designed Poxviral Promoters to Improve Immunogenicity and Efficacy of MVA-NP Candidate Vaccines against Lethal Influenza Virus Infection in Mice. Pathogens.

[179] Vatzia E., Feest K., McNee A., Manjegowda T., Carr B.V., Paudyal B., Chrun T., Maze E.A., Mccarron A., Morris S. (2023). Immunization with matrix-, nucleoprotein and neuraminidase protects against H3N2 influenza challenge in pH1N1 pre-exposed pigs. NPJ Vaccines.

[180] Villadiego J., García-Arriaza J., Ramírez-Lorca R., García-Swinburn R., Cabello-Rivera D., Rosales-Nieves A.E., Álvarez-Vergara M.I., Cala-Fernández F., García-Roldán E., López-Ogáyar J.L. (2023). Full protection from SARS-CoV-2 brain infection and damage in susceptible transgenic mice conferred by MVA-CoV2-S vaccine candidate. Nat. Neurosci..

[181] Russell M.S., Thulasi Raman S.N., Gravel C., Zhang W., Pfeifle A., Chen W., Van Domselaar G., Safronetz D., Johnston M., Sauve S. (2021). Single Immunization of a Vaccine Vectored by a Novel Recombinant Vaccinia Virus Affords Effective Protection Against Respiratory Syncytial Virus Infection in Cotton Rats. Front. Immunol..

[182] Endt K., Wollmann Y., Haug J., Bernig C., Feigl M., Heiseke A., Kalla M., Hochrein H., Suter M., Chaplin P. (2022). A Recombinant MVA-Based RSV Vaccine Induces T-Cell and Antibody Responses That Cooperate in the Protection Against RSV Infection. Front. Immunol..

[183] Alharbi N.K., Aljamaan F., Aljami H.A., Alenazi M.W., Albalawi H., Almasoud A., Alharthi F.J., Azhar E.I., Barhoumi T., Bosaeed M. (2022). Immunogenicity of High-Dose MVA-Based MERS Vaccine Candidate in Mice and Camels. Vaccines.

[184] Weskamm L.M., Fathi A., Raadsen M.P., Mykytyn A.Z., Koch T., Spohn M., Friedrich M., Bartels E., Gundlach S., Hesterkamp T. (2022). Persistence of MERS-CoV-spike-specific B cells and antibodies after late third immunization with the MVA-MERS-S vaccine. Cell Rep. Med..

[185] Chiuppesi F., Salazar M.D.A., Contreras H., Nguyen V., Martinez J., Park S., Nguyen J., Kha M., Iniguez A., Zhou Q. (2020). Development of a Multi-Antigenic SARS-CoV-2 Vaccine Using a Synthetic Poxvirus Platform. Res. Sq..

[186] Tscherne A., Hendrik Schwarz J., Rohde C., Kupke A., Kalodimou G., Limpinsel L., Okba N.M.A., Bošnjak B., Sandrock I., Odak I. (2021). Immunogenicity and efficacy of the COVID-19 candidate vector vaccine MVA-SARS-2-S in preclinical vaccination. Proc. Natl. Acad. Sci. USA.

[187] Mooij P., García-Arriaza J., Pérez P., Lázaro-Frías A., Verstrepen B.E., Böszörményi K.P., Mortier D., Fagrouch Z., Kiemenyi-Kayere G., Niphuis H. (2022). Poxvirus MVA Expressing SARS-CoV-2 S Protein Induces Robust Immunity and Protects Rhesus Macaques From SARS-CoV-2. Front. Immunol..

[188] Boulton S., Poutou J., Martin N.T., Azad T., Singaravelu R., Crupi M.J.F., Jamieson T., He X., Marius R., Petryk J. (2022). Single-dose replicating poxvirus vector-based RBD vaccine drives robust humoral and T cell immune response against SARS-CoV-2 infection. Mol. Ther..

[189] Pérez P., Lázaro-Frías A., Zamora C., Sánchez-Cordón P.J., Astorgano D., Luczkowiak J., Delgado R., Casasnovas J.M., Esteban M., García-Arriaza J. (2022). A Single Dose of an MVA Vaccine Expressing a Prefusion-Stabilized SARS-CoV-2 Spike Protein Neutralizes Variants of Concern and Protects Mice From a Lethal SARS-CoV-2 Infection. Front. Immunol..

[190] Perdiguero B., Marcos-Villar L., López-Bravo M., Sánchez-Cordón P.J., Zamora C., Valverde J.R., Sorzano C.Ó.S., Sin L., Álvarez E., Ramos M. (2023). Immunogenicity and efficacy of a novel multi-patch SARS-CoV-2/COVID-19 vaccine candidate. Front. Immunol..

[191] Kalodimou G., Jany S., Freudenstein A., Schwarz J.H., Limpinsel L., Rohde C., Kupke A., Becker S., Volz A., Tscherne A. (2023). Short- and Long-Interval Prime-Boost Vaccination with the Candidate Vaccines MVA-SARS-2-ST and MVA-SARS-2-S Induces Comparable Humoral and Cell-Mediated Immunity in Mice. Viruses.

[192] Wussow F., Kha M., Kim T., Ly M., Yll-Pico M., Kar S., Lewis M.G., Chiuppesi F., Diamond D.J. (2023). Synthetic multi-antigen MVA vaccine COH04S1 and variant-specific derivatives protect Syrian hamsters from SARS-CoV-2 Omicron subvariants. NPJ Vaccines.

[193] Ishigaki H., Yasui F., Nakayama M., Endo A., Yamamoto N., Yamaji K., Nguyen C.T., Kitagawa Y., Sanada T., Honda T. (2022). An attenuated vaccinia vaccine encoding the severe acute respiratory syndrome coronavirus-2 spike protein elicits broad and durable immune responses, and protects cynomolgus macaques and human angiotensin-converting enzyme 2 transgenic mice from severe acute respiratory syndrome coronavirus-2 and its variants. Front. Microbiol..

[194] Americo J.L., Cotter C.A., Earl P.L., Liu R., Moss B. (2022). Intranasal inoculation of an MVA-based vaccine induces IgA and protects the respiratory tract of hACE2 mice from SARS-CoV-2 infection. Proc. Natl. Acad. Sci. USA.

[195] Deschambault Y., Lynch J., Warner B., Tierney K., Huynh D., Vendramelli R., Tailor N., Frost K., Sajesh B., LeBlanc K. (2022). Single Immunization with Recombinant ACAM2000 Vaccinia Viruses Expressing the Spike and the Nucleocapsid Proteins Protects Hamsters against SARS-CoV-2-Caused Clinical Disease. J. Virol..

[196] Richert L., Lelièvre J.D., Lacabaratz C., Hardel L., Hocini H., Wiedemann A., Lucht F., Poizot-Martin I., Bauduin C., Diallo A. (2022). T Cell Immunogenicity, Gene Expression Profile, and Safety of Four Heterologous Prime-Boost Combinations of HIV Vaccine Candidates in Healthy Volunteers: Results of the Randomized Multi-Arm Phase I/II ANRS VRI01 Trial. J. Immunol..

[197] Rowland R., Pathan A.A., Satti I., Poulton I.D., Matsumiya M.M.L., Whittaker M., Minassian A.M., O’Hara G.A., Hamill M., Scott J.T. (2013). Safety and immunogenicity of an FP9-vectored candidate tuberculosis vaccine (FP85A), alone and with candidate vaccine MVA85A in BCG-vaccinated healthy adults: A phase i clinical trial. Hum. Vaccin. Immunother..

[198] Tameris M.D., Hatherill M., Landry B.S., Scriba T.J., Snowden M.A., Lockhart S., Shea J.E., McClain J.B., Hussey G.D., Hanekom W.A. (2013). Safety and efficacy of MVA85A, a new tuberculosis vaccine, in infants previously vaccinated with BCG: A randomised, placebo-controlled phase 2b trial. Lancet.

[199] Jordan E., Kabir G., Schultz S., Silbernagl G., Schmidt D., Jenkins V.A., Weidenthaler H., Stroukova D., Martin B.K., De Moerlooze L. (2023). Reduced Respiratory Syncytial Virus Load, Symptoms, and Infections: A Human Challenge Trial of MVA-BN-RSV Vaccine. J. Infect. Dis..

[200] Evans T.G., Bussey L., Eagling-Vose E., Rutkowski K., Ellis C., Argent C., Griffin P., Kim J., Thackwray S., Shakib S. (2022). Efficacy and safety of a universal influenza A vaccine (MVA-NP+M1) in adults when given after seasonal quadrivalent influenza vaccine immunisation (FLU009): A phase 2b, randomised, double-blind trial. Lancet Infect. Dis..

[201] Chiuppesi F., Zaia J.A., Frankel P.H., Stan R., Drake J., Williams B., Acosta A.M., Francis K., Taplitz R.A., Dickter J.K. (2022). Safety and immunogenicity of a synthetic multiantigen modified vaccinia virus Ankara-based COVID-19 vaccine (COH04S1): An open-label and randomised, phase 1 trial. Lancet Microbe.

[202] Hermann J.R., Fry A.M., Siev D., Slate D., Lewis C., Gatewood D.M. (2011). Stability of vaccinia-vectored recombinant oral rabies vaccine under field conditions: A 3-year study. Can. J. Vet. Res..

[203] Tomori O., Kolawole M.O. (2021). Ebola virus disease: Current vaccine solutions. Curr. Opin. Immunol..

[204] Moss B. (2013). Reflections on the Early Development of Poxvirus Vectors. Vaccine.

